# Diagnostic value of fat aspirates for amyloidosis in 950 patients

**DOI:** 10.1186/1750-1172-10-S1-P50

**Published:** 2015-11-02

**Authors:** JA Gilbertson, NA Botcher, D Rowczenio, C Whelan, HJ Lachmann, A Wechalekar, JD Gillmore, PN Hawkins

**Affiliations:** 1 National Amyloidosis Centre, UCL Division of Medicine, NW3 2PF, London, UK

## Background

Aspiration of abdominal subcutaneous fat is a simple outpatient procedure that is well established in management of patients with amyloidosis and is particularly useful when investigating the cause of organ dysfunction and more specifically in the context of a suspicion of amyloidosis. At the National Amyloidosis Centre (NAC), fat aspirates are routinely obtained in patients whom amyloid has not been previously confirmed. To date we have performed 950 fat aspirates to seek the presence of amyloid and identify the respective amyloid fibril protein. Thirty percent (290 cases) of the total aspirates sampled were found to contain amyloid. It was possible to conclusively type the fibril protein using immunohistochemistry (IHC) in 188 of these cases, thereby thwarting the need for a more invasive diagnostic biopsy or further diagnostic tests.

## Method

Abdominal fat tissue is aspirated and smears are prepared on to glass slides for Congo red staining. The remainder of the fat tissue aspirated is briefly fixed in formalin, double embedded in agar and then into a paraffin block (FFPE) for routine histology and IHC. Once amyloid has been confirmed IHC is performed using a panel of monospecific antibodies against known amyloid-forming proteins in an attempt to identify the amyloid fibril. Interpretation of all stained slides is carried out by two experienced people with and without crossed polarizing filters.

## Results

We found 97% concordance between the smear and the block results. Two percent of the smears gave a negative result for amyloid when the corresponding FFPE was positive, whereas 1% of the smears where positive when the FFPE was negative. Of the samples taken 60% did not contain any amyloid and 10% gave insufficient material for interpretation. Among the amyloidotic samples, IHC identified 25% as ATTR, 2% of other types (AA, AapoA1 and ALYS), 42% AL amyloid and 31% gave no immuno specific staining.

## Conclusion

Aspiration of abdominal subcutaneous fat is a valuable method in diagnosing amyloid, in 30% of cases preventing the need for a more invasive biopsy. There was a difference of 3% between the smear and the FFPE indicating that all representative tissue taken must be analysed to give a correct diagnosis. Only 42% of patients with negative samples went on to have a further tissue biopsy, over half of these samples did not contain any evidence of amyloid. Ten percent of the fat aspirates gave insufficient adipose material for interpretation, comprising mainly of blood. Immunohistochemical identification of the amyloid fibril protein was proven in 188 cases, in 31% of the cases the precise amyloid type was only determined after genetic sequencing. TTR by IHC was identified in 25% of the cases and among these patients, 62% were later found by genetic sequencing to have a variant, with V122l (p.V142l) being most common. Of the fat aspirates that did not demonstrate amyloid, only 6% of the patients were found to have a TTR variant.

